# Provoking a silent R gene in wheat genome confers resistance to powdery mildew

**DOI:** 10.1111/pbi.13903

**Published:** 2022-08-19

**Authors:** Miaomiao Li, Lei Dong, Keyu Zhu, Qiuhong Wu, Yongxing Chen, Ping Lu, Guanghao Guo, Huaizhi Zhang, Panpan Zhang, Beibei Li, Wenling Li, Yijun Yang, Yikun Hou, Xuejia Cui, Hongjie Li, Lingli Dong, Yusheng Zhao, Zhiyong Liu

**Affiliations:** ^1^ State Key Laboratory of Plant Cell and Chromosome Engineering, Institute of Genetics and Developmental Biology The Innovative Academy of Seed Design, Chinese Academy of Sciences Beijing China; ^2^ College of Advanced Agricultural Sciences University of Chinese Academy of Sciences Beijing China; ^3^ National Engineering Laboratory for Crop Molecular Breeding, Institute of Crop Sciences Chinese Academy of Agricultural Sciences Beijing China; ^4^ Hainan Yazhou Bay Seed Laboratory Sanya City China

**Keywords:** *Pm41*, allelic variation, genetic diversity, transposon, gene silencing

Common wheat (*Triticum aestivum*. L) is one of the most widely cultivated staple crops in the world (Tadesse *et al*., 2019), and it has been always important to breed pathogen‐resistant varieties for safeguarding its production (Singh *et al*., 2016). However, common wheat genetic diversity has been narrowed throughout its entire existence due to two sequential polyploidization events followed by domestication (IWGSC, 2014), therefore profoundly limiting its improvement like pathogen resistance. Here, we provide an alternative route to increase the genetic diversity toward future wheat breeding by exploiting the silent genetic loci hidden in the wheat genome.

Powdery mildew, caused by the fungus *Blumeria graminis* f. sp. *tritici* (*Bgt*), is a severe foliar disease of wheat causing reduction in grain yield and quality (Savary *et al*., 2019). Host resistance is widely considered the first and most effective barrier of defence that prevents the invasion of the pathogen (Wu *et al*., 2021). We previously isolated the powdery mildew resistance gene *Pm41* (hereof *Pm41a*), which encodes a typical coiled‐coil, nucleotide‐binding site, and leucine‐rich repeat protein (CNL) from wild emmer wheat (WEW, *Triticum dicoccoides*) accession ‘IW2’ (Li *et al*., 2020). Moreover, three *Pm41* haplotypes including Hap1 (*Pm41a*, ‘IW2’), Hap2 (‘Langdon’, LDN’), and Hap3 (null, ‘Chinese Spring’) were identified in diversified worldwide wheat collection with a *Pm41a* gene‐specific marker *WGGB427*. To further characterize the natural diversity of *Pm41*, the entire locus of *Pm41* was amplified with overlapping gene‐specific primers (Table S1) in a representative diversified panel including 131 WEW, 38 durum wheat (*T. durum* Desf.), and 31 common wheat (Table S2), resulting in the identification of seven haplotypes (Figure 1a). *Pm41a*, Hap2, Hap3, and four new haplotypes (Hap4 to Hap7) were identified in the WEW populations (Figures 1b and S1). In contrast, only three haplotypes (Hap2, Hap3, and Hap7) exist in durum and hexaploid wheat populations, suggesting loss of genetic diversity of the *Pm41* locus due to domestication and polyploidization bottleneck. Hap2 (hereof *Pm41b*) only accounts for 5% of the WEW but was predominant in those tested accessions of durum (90%) and hexaploid (90%) wheats (Figure 1b and Table S2). Detailed analysis showed that *Pm41b* contains an intact *Pm41a* allelic coding region sequence (Figure S1) but carries two DNA transposons inserted in the promoter and 3′‐UTR regions (Figures 1c and S2). Different from the *Pm41a* allele, which is resistant and well‐induced upon *Bgt* isolate E09 inoculation in the highly resistant WEW accession ‘IW2’ (Figure 1d–f; Li *et al*., 2020), *Pm41b* shows no expression before or after *Bgt* isolate E09 inoculation in the highly susceptible tetraploid durum wheat cultivar ‘LDN’ (Figure 1d–f). Furthermore, a similar result was obtained in the susceptible common wheat cultivar ‘Fielder’ (Figure 1f) carrying the same *Pm41b* allele as ‘LDN’ (Table S2), suggesting that *Pm41b*, as well as other *Pm41* haplotypes (Figure S2), are silenced alleles of *Pm41a*, probably due to the transposon insertions and represent unexploited hidden variations for powdery mildew resistance in modern wheat breeding.

**Figure 1:**
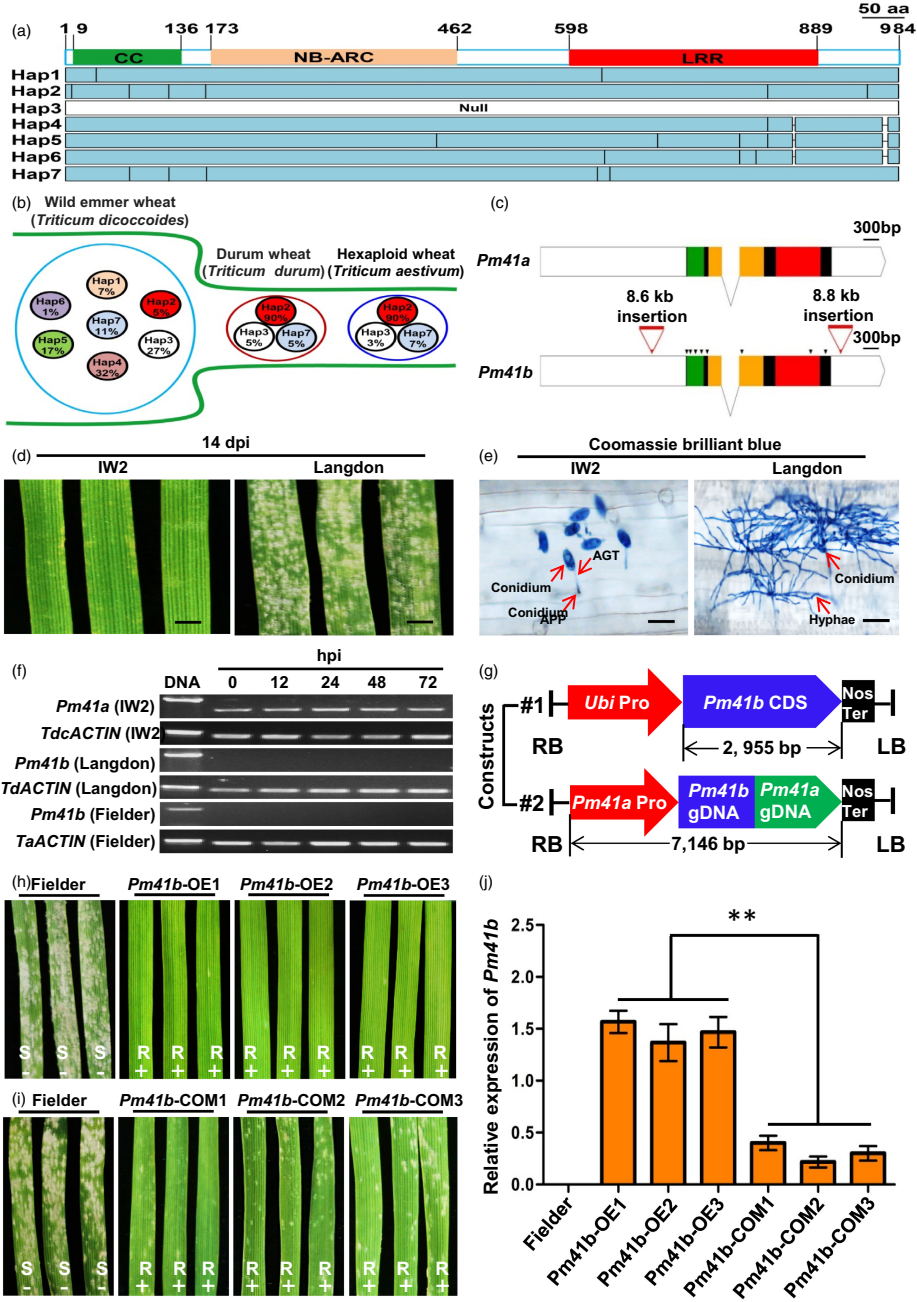
Provoking a silent *Pm41b* gene conferring powdery mildew resistance. (a) Haplotype analysis of *Pm41* alleles. (b) Genetic bottleneck of *Pm41* alleles from wild emmer wheat to domesticated durum and bread wheat. (c) Gene structure and variations of *Pm41a* and *Pm41b*. Green, yellow, and red rectangles represent predicted coiled‐coil, NB‐ARC, and LRR domain, respectively. Black triangles show the nucleotide (amino acid) differences between *Pm41a* and *Pm41b*. (d) Leaves at 2‐leaf‐stage of wild emmer wheat accession ‘IW2’ and durum wheat ‘Langdon’ challenged with *Bgt* isolate E09 at 14 day post‐inoculation (dpi). Scale bar, 3 mm. (e) Fungal structures of *Bgt* isolate E09 at 5 dpi as stained by Coomassie brilliant blue. AGT, appressorial germ tube; APP, appressorium. Scale bar, 100 μm. (f) RT‐PCR analysis of *Pm41a* and *Pm41b* in ‘IW2’, ‘Langdon’ and ‘Fielder’. The *TdcACTIN*, *TdACTIN* and *TaACTIN* genes from IW2, Langdon and Fielder, respectively, were used as internal controls. (g) Schematic diagram of Pro*Ubi*:*Pm41b* and Pro*Pm41a*:*Pm41b* used for transformation of powdery mildew susceptible cv. ‘Fielder’. Pro*Pm41a*:*Pm41b* includes a 2388 bp presumed native promoter of *Pm41a*, the 3370 bp entire genomic sequence of *Pm41b* including potentially coding and intron regions, and a 1390 bp terminator region of *Pm41a*. Ubi, promoter of the maize ubiquitin gene. (h) Infection reactions of T_1_ transgenic plants of Pro*Ubi*:*Pm41b* and (i) Pro*Pm41a*:*Pm41b*. ‘Fielder’ was used as a susceptible control. Three individuals of three independent families are shown. The “+”and “−” signs designate the presence or absence of *Pm41b* transgenes. (j) The transcript levels of *Pm41b* in T_2_ transgenic plants of Pro*Ubi*:*Pm41b* and Pro*Pm41a*:*Pm41b*. Error bars represent ± SEMs of three independent experiments. Statistically significant differences (Student's *t*‐test): **, *P* < 0.01.

To further explore the functionality of *Pm41b in vivo*, as well as its possibility of application in wheat breeding, we therefore generated two types of transgenic wheat plants (Ishida *et al*., 2015) carrying *Pm41b* driven either by the *Pm41a* promoter or by the constitutively expressing promoter of ubiquitin from maize (*Zea mays* L.) (Figure 1g). The T_0_ and T_1_ seedlings from both types of transgenic plants were challenged with *Bgt* isolate E09. The transgenic plants carrying the ubiquitin‐driven *Pm41b* gene were immune to isolate E09 with infection types (ITs) of 0 (immune) ‐ 0; (necrotic fleck) (Figures 1h, S3 and Table S3), while the other transgenic plants carrying *Pm41b* driven by the native promoter of *Pm41a* were resistant to isolate E09 with IT of 1 (highly resistant) (Figure 1i and Table S3). Moreover, co‐segregation of the *Pm41b* transgene with powdery mildew resistance was observed in the T_2_ segregating progenies (Table S3), supporting the functionality of *Pm41b* in powdery mildew resistance. The transcript level of *Pm41b* in the transgenic plants carrying the ubiquitin‐driven *Pm41b* gene is about three folds higher than that in the transgenic plants carrying *Pm41b* driven by the *Pm41a* native promoter (Figure 1j), in correlation to their resistance, suggesting *Pm41b* may function in a dosage‐dependent way. In addition, the increased powdery mildew resistance of *Pm41b‐OE* and *Pm41‐COM* transgenic plants did not impact on major agronomic traits (Figure S4). The abovementioned results imply that the silent genetic loci like *Pm41b* are a valuable resource of genetic variation in the wheat genome and therefore could be potentially utilized to enrich the genetic diversity in wheat breeding.

The genomes of wheat, as well as other crops, contain a large proportion of inactive or silenced genetic loci, many of which are related to the key agronomic traits and stress resistance and would enormously contribute to the pool of genetic variation if properly modulated. For instance, about 84% of wheat genome are transposon elements (IWGSC, 2014), exhibiting the significance of provoking those silenced loci due to interference by the transposon elements. In this study, we examined this idea by provoking a silent *Pm41b* with a functional *Pm41a* promoter, which surprisingly conferred sound powdery mildew resistance in hexaploid wheat. Our work sheds a light on how to wake up and make use of those ‘sleeping beauties’ in the wheat genome. It provides an intriguing approach to exploit genetic diversity, which is extremely narrowing and being a barrier of crop improvement in modern cultivars. The emergence of precise genome editing tools will offer more efficient approaches to directly modulate these silent but useful loci in modern cultivars and booster the modern crop breeding for food sustainability.

## Conflicts of interest

The authors declare no conflict of interest.

## Author contributions

Z.L., Y.Z., and L.L.D. conceived the study. M.L., L.D., K.Z., and X.C. performed the experiments. H.Z., G.G., and P.Z. contributed bioinformatic analysis. L.L.D., Q.W., Y.C., and P.L. participated in the transgene experiment. B.L., W.L., Y.Y., and Y.H. did the sampling for DNA and RNA extraction and tested the resistance to powdery mildew. Z.L., Y.Z., M.L., and H.L. wrote the manuscript.

## Accession number

The sequences of the *Pm41* alleles are available in GenBank (accession numbers: ON059149‐ON059153).

## Supporting information


**Figure S1** Multiple sequence alignment of Pm41 haplotypes.
**Figure S2** Expression and genomic analysis of *Pm41* alleles.
**Figure S3** Fungal structures of *Bgt* isolate E09 at 5 dpi as stained by Coomassie brilliant blue.
**Figure S4** Statistical analysis of plant height (a), tiller number (b), flag leaf length (c), flag leaf width (d), ear length (e), grain number per ear (f), thousand‐grain weight(g) (g), and grain weight per plant (h) in Fielder, Pm41b‐OE1, Pm41b‐OE2, Pm41b‐OE3, Pm41b‐COM1, Pm41b‐COM2 and Pm41b‐COM3 plants.

**Table S1** Primers used in the study.
**Table S2** Haplotypes of Pm41 in wheat accession tested.
**Table S3** Powdery mildew infection assays on Pm41b transgenic plants.
