# Supplementary material for: Provoking a silent R gene in wheat genome confers resistance to powdery mildew
Source: Plant Biotechnol J. 2022 Aug 19;20(11):2039–41. doi: 10.1111/pbi.13903 (PMC9616516; doi:10.1111/pbi.13903)
Supplement: Supplementary file 1 — Figure S1 Multiple sequence alignment of Pm41 haplotypes. Figure S2 Expression and genomic analysis of Pm41 alleles. Figure S3 Fungal structures of Bgt isolate E09 at 5 dpi as stained by Coomassie brilliant blue. Figure S4 Statistical analysis of plant height (a), tiller number (b), flag leaf length (c), flag leaf width (d), ear length (e), grain number per ear (f), thousand‐grain weight(g) (g), and grain weight per plant (h) in Fielder, Pm41b‐OE1, Pm41b‐OE2, Pm41b‐OE3, Pm41b‐COM1, Pm41b‐COM2 and Pm41b‐COM3 plants. [file PBI-20-2039-s002.pptx]

## Slide 1
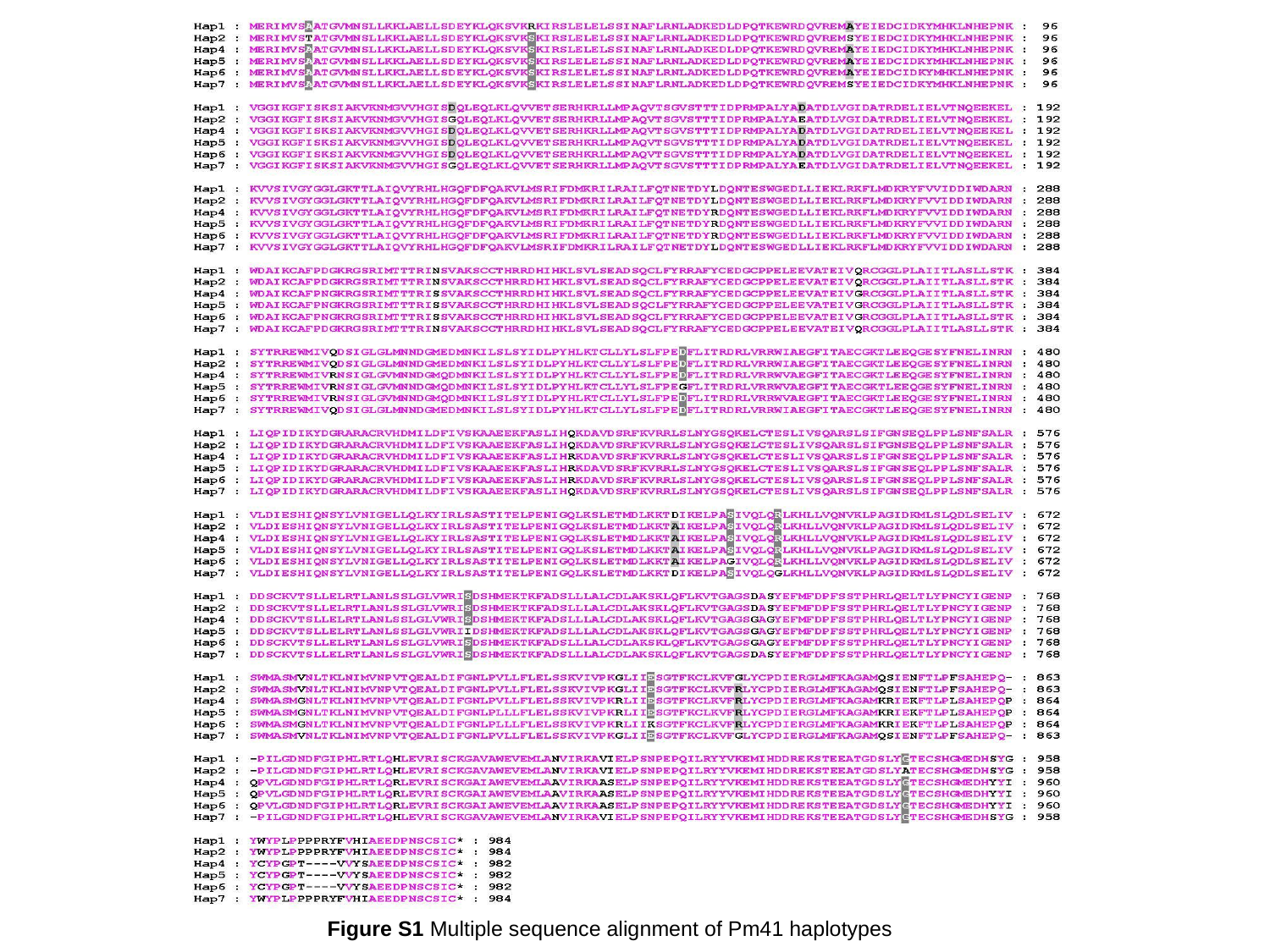

Figure S1 Multiple sequence alignment of Pm41 haplotypes

## Slide 2
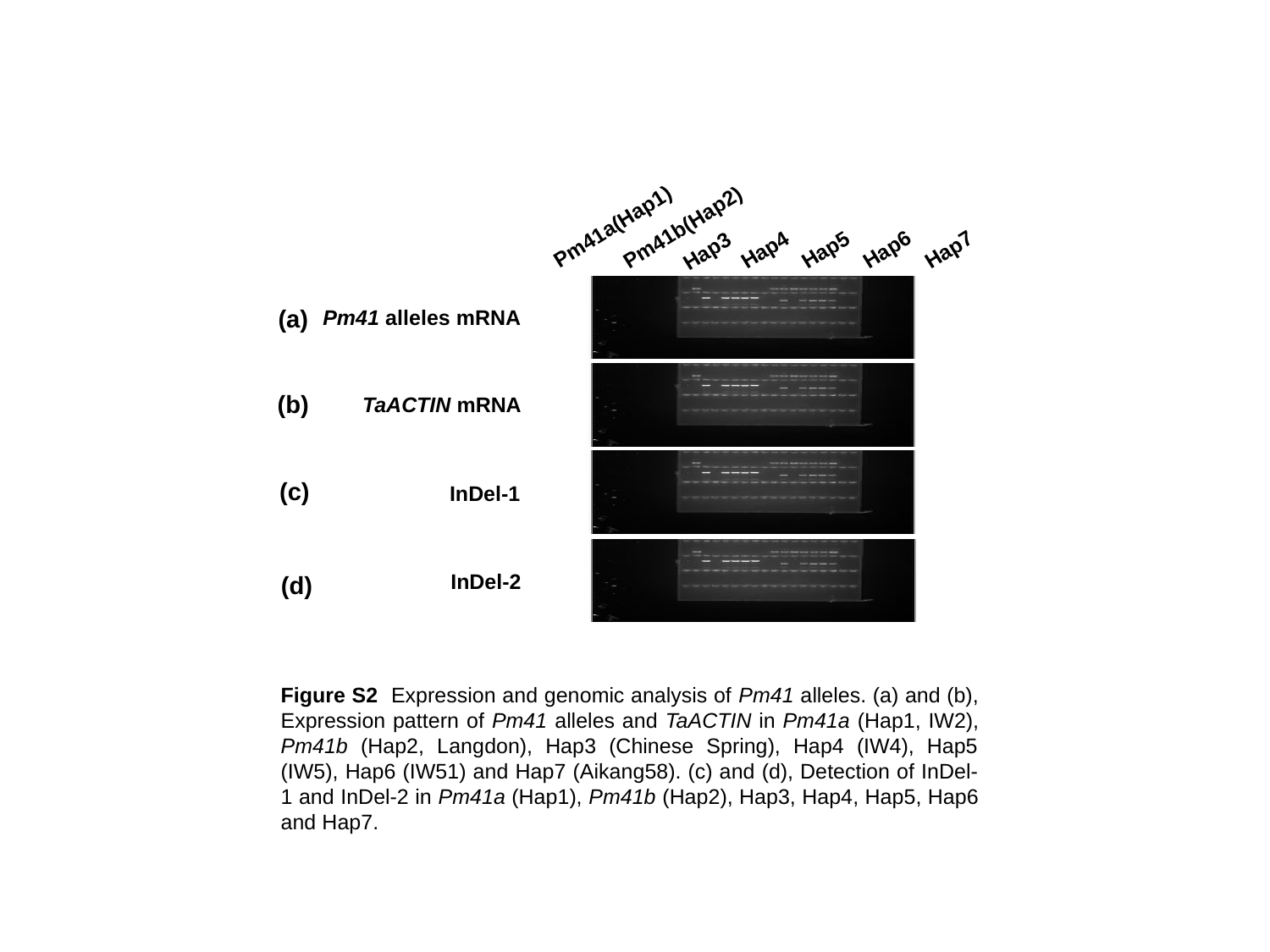

Pm41a(Hap1)
Pm41b(Hap2)
Hap6
Hap7
Hap5
Hap4
Hap3
(a)
Pm41 alleles mRNA
(b)
TaACTIN mRNA
(c)
InDel-1
InDel-2
(d)
Figure S2 Expression and genomic analysis of Pm41 alleles. (a) and (b), Expression pattern of Pm41 alleles and TaACTIN in Pm41a (Hap1, IW2), Pm41b (Hap2, Langdon), Hap3 (Chinese Spring), Hap4 (IW4), Hap5 (IW5), Hap6 (IW51) and Hap7 (Aikang58). (c) and (d), Detection of InDel-1 and InDel-2 in Pm41a (Hap1), Pm41b (Hap2), Hap3, Hap4, Hap5, Hap6 and Hap7.

## Slide 3
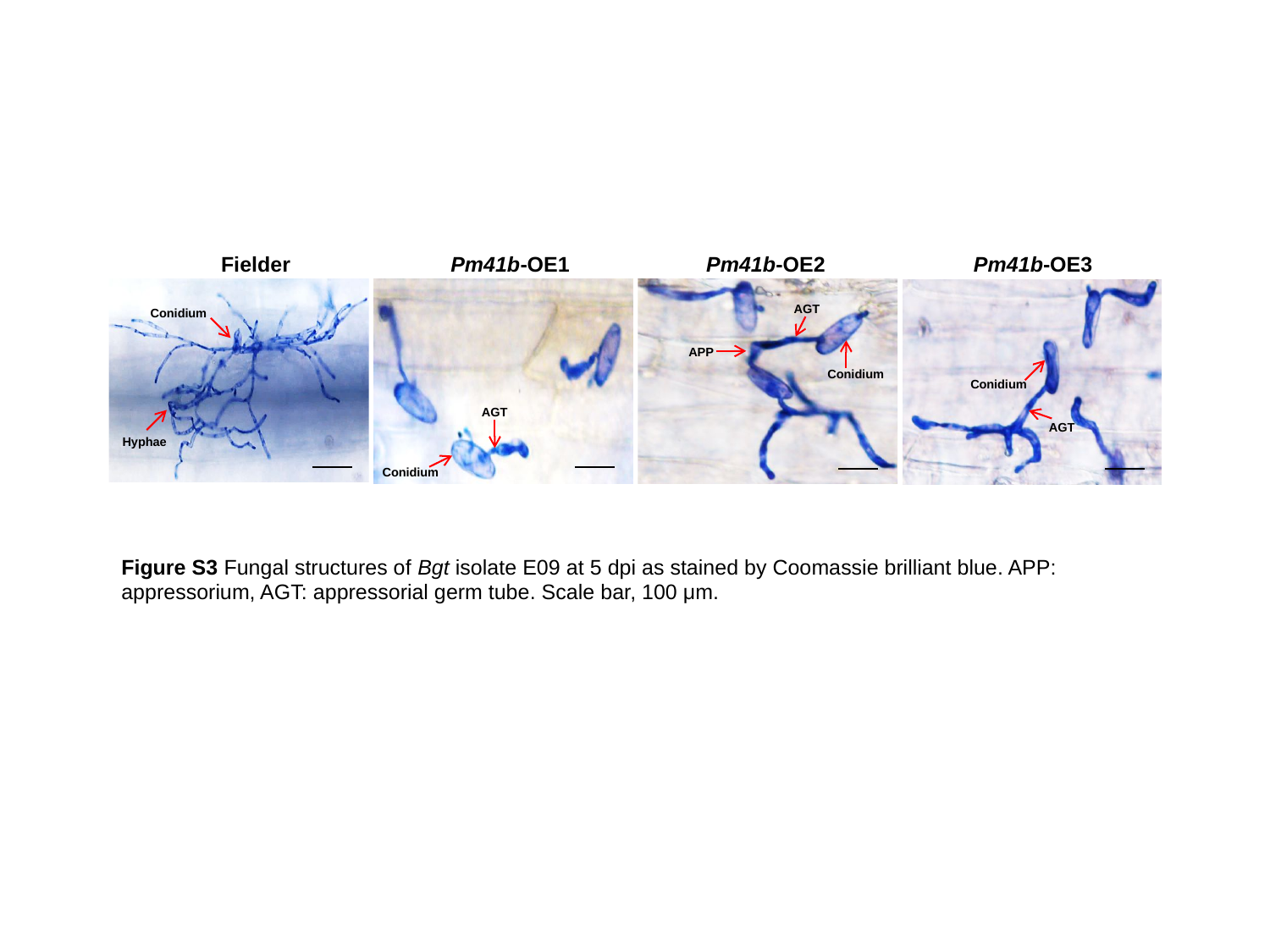

Fielder Pm41b-OE1 Pm41b-OE2 Pm41b-OE3
AGT
Conidium
APP
Conidium
Conidium
AGT
AGT
Hyphae
Conidium
Figure S3 Fungal structures of Bgt isolate E09 at 5 dpi as stained by Coomassie brilliant blue. APP: appressorium, AGT: appressorial germ tube. Scale bar, 100 μm.

## Slide 4
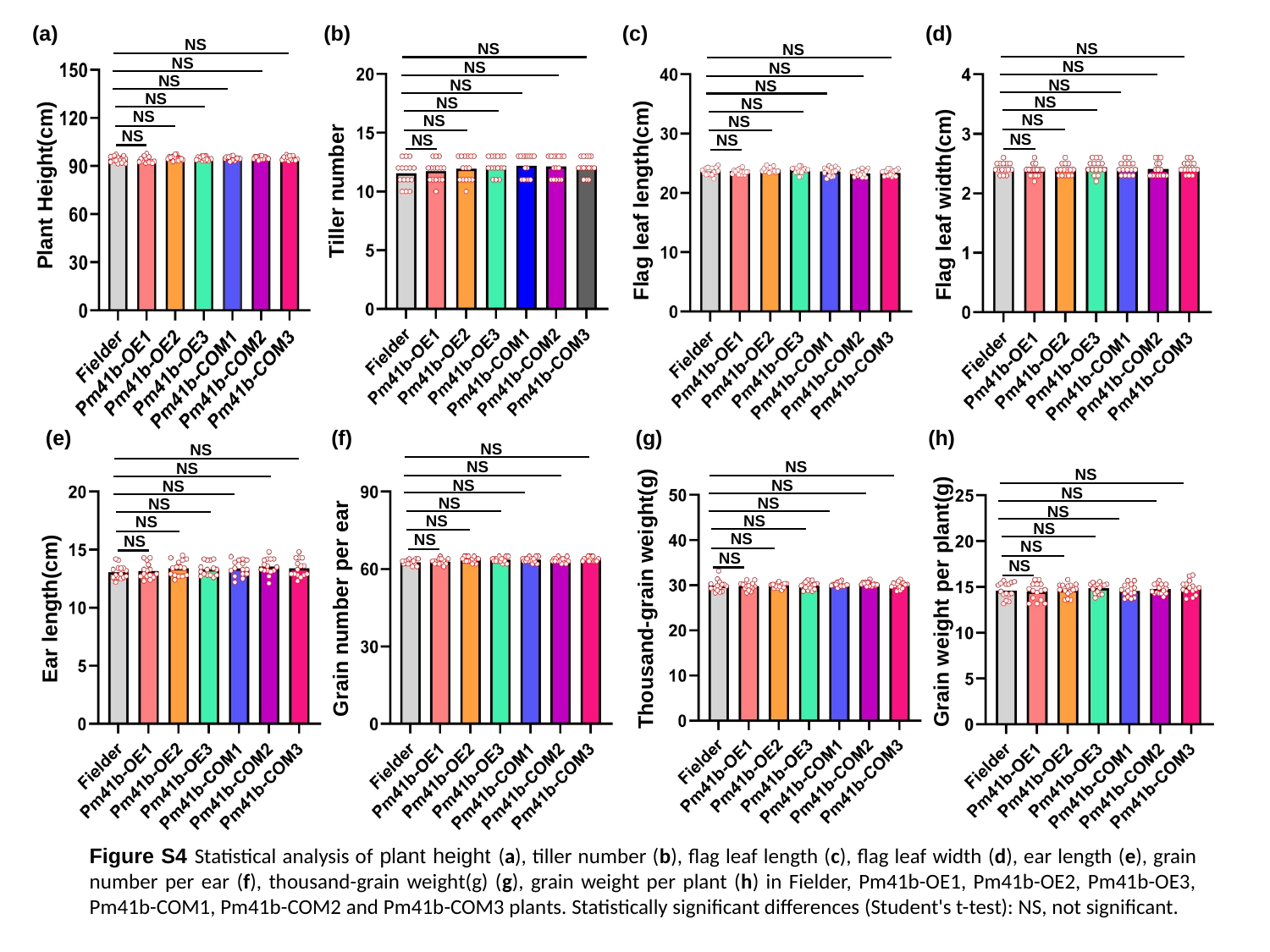

(a) (b) (c) (d)
NS
NS
NS
NS
NS
NS
NS
NS
NS
NS
NS
NS
NS
NS
NS
NS
NS
NS
NS
NS
NS
NS
NS
NS
Plant Height(cm)
Tiller number
Flag leaf length(cm)
Flag leaf width(cm)
(e) (f) (g) (h)
NS
NS
NS
NS
NS
NS
NS
NS
NS
NS
NS
NS
NS
NS
NS
NS
NS
NS
NS
NS
NS
NS
NS
NS
Thousand-grain weight(g)
Grain weight per plant(g)
Grain number per ear
Ear length(cm)
Figure S4 Statistical analysis of plant height (a), tiller number (b), flag leaf length (c), flag leaf width (d), ear length (e), grain number per ear (f), thousand-grain weight(g) (g), grain weight per plant (h) in Fielder, Pm41b-OE1, Pm41b-OE2, Pm41b-OE3, Pm41b-COM1, Pm41b-COM2 and Pm41b-COM3 plants. Statistically significant differences (Student's t-test): NS, not significant.
